# The effects of height-for-age and HIV on cognitive development of school-aged children in Nairobi, Kenya: a structural equation modelling analysis

**DOI:** 10.3389/fpubh.2023.1171851

**Published:** 2023-06-21

**Authors:** Rachel Maina, Jia He, Amina Abubakar, Miguel Perez-Garcia, Manasi Kumar, Jelte M. Wicherts

**Affiliations:** ^1^Department of Methodology and Statistics, Tilburg University, Tilburg, Netherlands; ^2^Brain and Mind Institute, Aga Khan University, Nairobi, Kenya; ^3^Neurosciences Unit, KEMRI-Wellcome Trust Research Programme, Nairobi, Kenya; ^4^Institute for Human Development, Aga Khan University, Nairobi, Kenya; ^5^Mind, Brain and Behaviour Research Center (CIMCYC), University of Granada, Granada, Spain; ^6^Department of Psychiatry, University of Nairobi, Nairobi, Kenya

**Keywords:** stunting, mediation, HIV, lower school students, executive functioning, reasoning, flexibility, lower & middle-income countries

## Abstract

**Background:**

Empirical evidence indicates that both HIV infection and stunting impede cognitive functions of school-going children. However, there is less evidence on how these two risk factors amplify each other’s negative effects. This study aimed to examine the direct effects of stunting on cognitive outcomes and the extent to which stunting (partially) mediates the effects of HIV, age, and gender on cognitive outcomes.

**Methodology:**

We applied structural equation modelling to cross-sectional data from 328 children living with HIV and 260 children living without HIV aged 6–14 years from Nairobi, Kenya to test the mediating effect of stunting and predictive effects of HIV, age, and gender on cognitive latent variables flexibility, fluency, reasoning, and verbal memory.

**Results:**

The model predicting the cognitive outcomes fitted well (RMSEA = 0.041, CFI = 0.966, χ^2^ = 154.29, DF = 77, *p* < 0.001). Height-for-age (a continuous indicator of stunting) predicted fluency (*β* = 0.14) and reasoning (*β* = 0.16). HIV predicted height-for-age (*β* = −0.24) and showed direct effects on reasoning (*β* = −0.66), fluency (*β* = −0.34), flexibility (*β* = 0.26), and verbal memory (*β* = −0.22), highlighting that the effect of HIV on cognitive variables was partly mediated by height-for-age.

**Conclusion:**

In this study, we found evidence that stunting partly explains the effects of HIV on cognitive outcomes. The model suggests there is urgency to develop targeted preventative and rehabilitative nutritional interventions for school children with HIV as part of a comprehensive set of interventions to improve cognitive functioning in this high-risk group of children. Being infected or having been born to a mother who is HIV positive poses a risk to normal child development.

## Introduction

Stunting (a height-for-age Z score of below −2 SD) (1) affects more than 149.2 million children worldwide and is associated with cognitive impairment (2–4) linked to poor academic performance (5, 6). Children who are stunted are at risk of underperforming in school and consequently dropping out (7). Over time, decreased years of education may result in low intelligence or cognitive ability (8). These may further contribute to long-term effects of reduced income and increased poverty (1, 7, 9, 10). Indeed Hoddinott et al. (10) found that stunting at 2 years was associated with increased probability of poverty in adulthood. Stunting has also been associated with increased mortality, morbidity, and a vicious cycle of stunting between mothers and children if left unaddressed (3, 9). This cycle is characterized by stunted mothers who tend to have a higher probability of lower age at first birth and multiple births (10) leading to increased nutritional demands on the mother (3); if not met, may lead to undernutrition in children (11). Moreover, mothers with a history of stunting, are likely to have short stature/adult height (7) which is linked to obstetric complications during birth and having children with small gestational age (SGA) (7, 9). SGA has been associated with up to 20% of stunting in children under the age of 5 years (9). This cyclical disability effects of stunting have attracted worldwide attention with underlying factors such as poverty and hunger forming part of the amelioration efforts in the sustainable development goals (12). These two factors have been incorporated as targets of intervention given the proven association between stunting and poverty (7, 10), and hunger resulting in deficient diets that do not meet the nutritional standards needed to prevent stunting (11). These primarily nutrition-specific interventions have achieved population-wide traction and success in reducing stunting. For example, countries have already put in place measures to curtail stunting that have borne fruits, with Asian countries showing a stunting decrease from 49 to 28% between 1990 and 2010. However, in Africa stunting has remained stagnant at around 40% (3). To achieve results similar to those achieved in Asia, nutrition-specific interventions have been primarily advocated assuming that they will reverse the effects of stunting (9). However, an increase in height does not necessarily mean that the child’s cognitive function is restored and working according to age. Nutrition may increase a child’s height but not necessarily ameliorate cognitive impairment post stunting because other factors may also impair cognitive functioning in a child with stunting. Specifically, other factors such as HIV infection, poverty, and poor health may affect cognitive outcomes, particularly in low and middle-income countries (LMICs) (13). Children with short stature (having a height that is well below that of other children of the same age and sex) may exhibit poor or delayed cognitive development for various reasons. For example, stunting is highly prevalent among children with HIV (28.6% in Kenya) (14) and children who are both HIV positive and stunted could have worse cognitive outcomes. Investigating causal mechanisms between stunting and cognitive performance may inform the alignment of stunting interventions to programmatic goals for the comprehensive management of HIV for school-going children.

## HIV, stunting, and cognitive development

Given normal cognitive development, children’s cognitive functioning develops because of environmental factors and brain myelination among other neurological mechanisms and other factors involved in cognitive development (15, 16). HIV has been found to negatively impact cognitive function (17), and stunting (18) may partially explain this link. HIV is neurotropic meaning it directly affects the central nervous system (CNS) which may lead to cognitive impairment (19). Indirectly, HIV infection puts children at risk of undernutrition through inadequate and imperfect absorption of food, opportunistic infections, some HIV drugs, and other aetiological factors (20). Chronic undernutrition manifests itself as stunting. Children who are stunted have been found to perform poorly in receptive vocabulary and numerical ability compared to children who are not stunted, whereas children infected with HIV perform poorly in receptive and expressive language and attention compared to those without HIV (17, 21). Children with HIV are also found to have poorer cognitive performance in draw-a-person task and digit span (22), and working memory and executive functioning (23) though some studies have not found any difference in general cognitive function (24). The few earlier studies on both HIV and stunting among school-age children or lower school students used only a partial set of cognitive functions (25, 26). Stunting in children has been found to predict performance in reasoning, memory, language, executive functions, and motor ability (25, 26) while HIV predicts performance in nonverbal cognitive abilities, executive function, processing speed, memory, planning, reasoning, working memory, and visual–spatial abilities (22, 27, 28). These cognitive functions fall short of the recommended assessment domains (29, 30) of memory, language, attention, perceptual-motor, executive function, and social cognition (30). Therefore, more research using a broad battery of tests could shed light on how both HIV and stunting affect cognitive development.

We study the predictive effects (in relation to our model) of stunting and HIV on cognitive outcomes, while also considering age (31–33) and gender (15, 34–37) as relevant factors in predicting both stunting and cognitive outcomes. Age is central to stunting because the definition of stunting includes height-for-age ratio (1). Moreover, cognitive performance normally increases with age right from birth (16, 26, 38), although the developmental trajectories might vary over cognitive functions (34, 39) and might differ between children (40) for a host of reasons such as nutrition, exposure to HIV, parental education, and parental income (13, 22, 25, 26). Gender is relevant to our understanding of the effects of HIV and stunting on cognitive development because gender differences that vary in strength and direction have previously been found in relatively healthy children populations (15, 34, 35).

In this study, we investigate the mediation effects of stunting (as measured by height for age) of the link between HIV, age, and gender on cognitive functions recommended for assessment in the diagnostic statistical manual of mental disorders version five (DSM V) among school-age children from Kenya. The DSM V recommended classification of neurocognitive domains was preferred in this study due to the domains consistency with available knowledge on etiology of neurocognitive disorders and their impaired cognitive functions and with assessment criteria developed by experts (41, 42). We also prefer DSM V criteria because we hope that the findings can inform a holistic approach to clinical management of children with HIV. We hypothesized that stunting would partially mediate the effects of HIV on cognitive outcomes among 6-14-year-olds. The study used the Computerised Battery for Neuropsychological Evaluation of Children (BENCI) (43) – a cognitive battery that has good validity and reliability for diverse cultures including low-income settings and that can measure the cognitive functions recommended in DSM V. The outcome of this study may shed light on which cognitive domains are most impacted by stunting within a population infected with HIV to inform future interventions for improving cognitive functioning.

## Methods

### Design and setting

We evaluated the effects of stunting and HIV status on cognitive functions in a cross-sectional case control study among 6 to 14-year-olds within an HIV programme and three public schools. This study was part of a larger study that validated the Computerised Battery for Neuropsychological Evaluation of Children (BENCI) in Kenya (19).

The HIV uninfected sample was taken from three public primary schools in a middle-class urban setting. The schools follow the Kenyan government structured curriculum where children aged 6 years are in grade 1. The case sample was taken from a HIV programme in a middle-class urban setting. The programme provides a community based intervention to address medical, social, and economic needs of HIV positive children and their families. Both study settings are in Kenya’s capital city, Nairobi. Nairobi’s population is above the national poverty average (36.1%) and also above the national severe stunting average (11.4%) (44). Nairobi’s food consumption relies heavily on food production from other regions within the country and its inhabitants spend more on food than those in rural regions with the major food category being cereals (45).

### Ethics approval

The study received ethics approval from Tilburg University’s School of Humanities Research Ethics Committee (REC# 2017/25) and the Kenyatta National Hospital/ University of Nairobi Ethical Review Committee (P556/07/2016). Additional approvals were sought from the County Government of Nairobi. Heads of the study sites authorized the study, while the caregivers gave informed consent and the children gave assent after a careful explanation of what the study entailed.

### Study sample characteristics

Children who met the eligibility criteria were recruited into the study. The inclusion criteria were: all children aged 6–14 years and, for the control group, not having any medical condition as reported by the school and the students themselves, while for the experimental group, not having comorbid conditions as reported by caregivers and children themselves. We excluded children with comorbid and/or severe medical conditions associated with being HIV-positive as indicated in their medical reports. Children were recruited from four clinics within the HIV programme and three public primary schools. In the clinics, the staff helped in generating a database of children who met the inclusion criteria, and we aligned our recruitment process to their next hospital visit, which was also a play day for the children and fell on a weekend. On attending their scheduled clinic appointment, the parents of potential participants were randomly identified and informed about the study while being requested to sign up for the study. In the school setting, the same procedure was undertaken though the teachers here helped in randomly selecting the students who met inclusion criteria. Language wise, the Kenyan government obligates parents to send all children to school. The language of instruction in the schools is English, although the children prefer to use Kiswahili, Kenya’s national language, in their daily communication.

### Data collection procedure

The data collection was conducted by clinical psychologists. Once consent was given, the children were immediately shown to a room in which data collectors designated them to a table with an iPad. After anthropometric measures were taken, children completed the cognitive assessment using the BENCI on the iPads, which took around 90 to 120 min. There was a 10-min break between the BENCI subtests which was scheduled right after the sustained attention subtest which can be tedious for children.

### Measures

The computerised BENCI has been adapted and validated for use among Kenyan children aged 6–14 years in urban settings (19, 43) and has 17 tests that measure the following: processing speed, motor coordination, attention (sustained and selective), memory (verbal and visual), language (comprehension and production), and executive function (updating/monitoring, inhibition/impulsivity, flexibility, working memory, planning) (46). A detailed description of the tests, their administration, and scoring has been written about elsewhere (19, 43). Using the same data as reported here, BENCI has been found to have good test–retest reliability for most subtests and sufficient internal consistencies ranging from 0.50 to 0.97 (19). A four-factor model consisting of flexibility, fluency, reasoning, and verbal memory fitted the data well (RMSEA = 0.052, CFI = 0.944, TLI = 0.914) and showed metric (RMSEA = 0.040, CFI = 0.930, TLI = 0.893) and partial scalar measurement invariance (RMSEA = 0.041, CFI = 0.920, TLI = 0.884) between the HIV positive and negative groups (19). The tool has shown convergent validity with reasoning, memory, and inhibition tests from a local test battery called the Kilifi Toolkit (19). The BENCI was ideal for this study because it integrates tests that measure neurocognitive indicators recommended in DSM V and its good psychometric properties in our setting. We collected socio-demographic information about age, gender, weight, and height. Age was determined from the year of birth and calculated in terms of complete years and months since birth. Weight in kilograms was measured by a body scale as per the WHO protocol (47), and height in meters was measured by a tape measure.

Age was measured as complete years while gender was measured as either male or female.

### Analyses

Stunting was calculated using the height-for-age z score (HAZ) based on 5–19 year-olds’ WHO Child Growth Reference standards where age was calculated in months (48). A WHO developed syntax was used to compute height-for-age (48). Children who were not stunted were defined as having a HAZ of ≥ −2.0 SD, those moderately stunted scored <−2.0 SD to > −3.0 SD, while those that were severely stunted scored ≤ −3.0 SD (48). The analyses used a continuous variable defined as height-for-age z score to measure stunting, with lower z values indicating more stunting.

**Figure 1:**
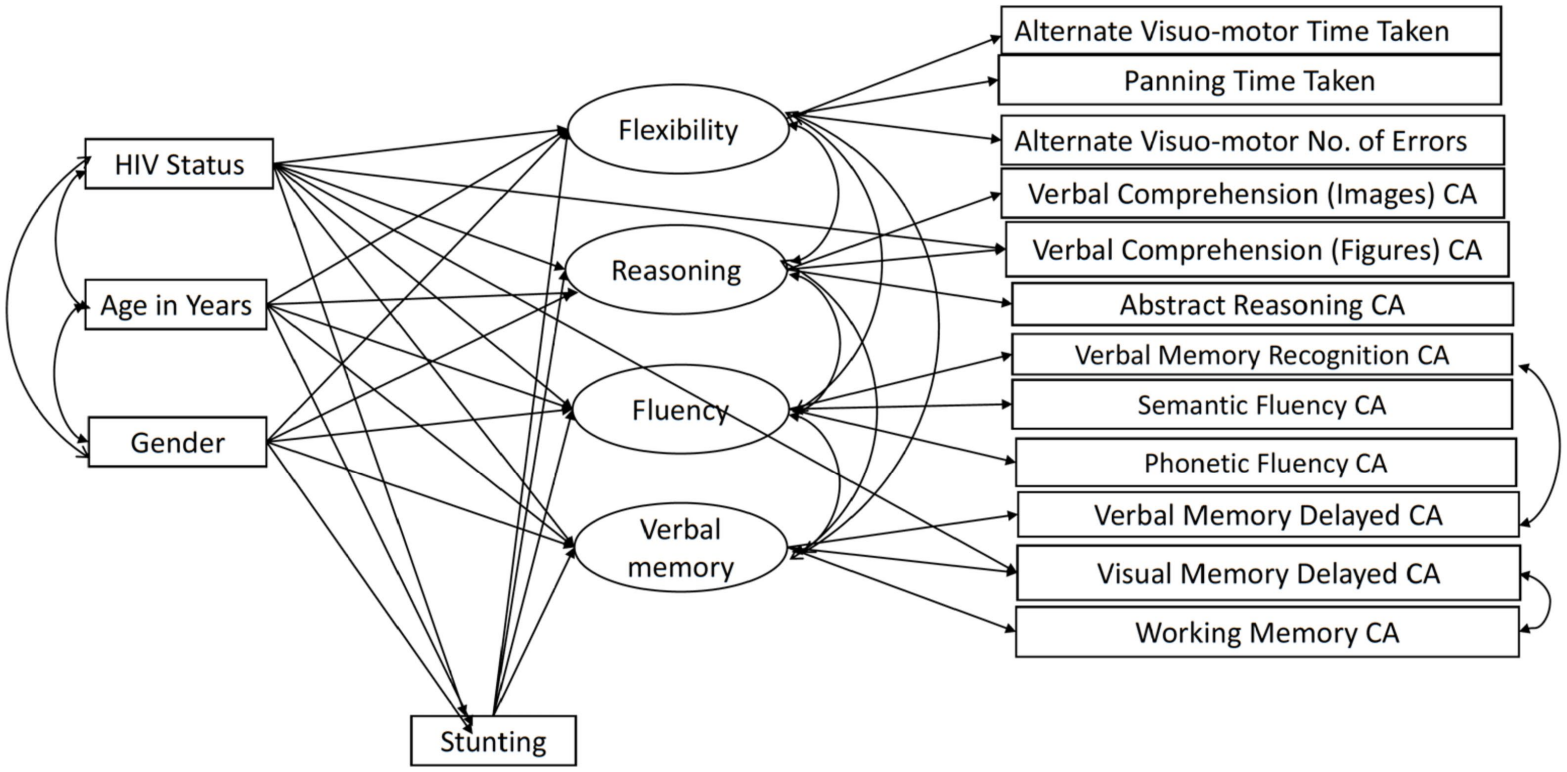
The BENCI measurement model with adapted partial scalar invariance and modification index paths. CA, Correct Answers.

We used maximum likelihood estimation in AMOS (49) to fit a structural equation model (SEM) as depicted in Figure 1. We used SEM as opposed to a multivariate path analysis as SEM allowed us to estimate a well-fitting complex model featuring latent cognitive variables underlying subtest scores (50). The model tests the mediating effect of height-for-age and includes all direct effects of HIV, age, and gender on the cognitive latent variables. The measurement model for the BENCI had been previously confirmed in another study using the same data (19). We adapted the model a bit due to the partial scalar invariance findings. The adaptation involved additional direct paths from HIV to Verbal Comprehension Figures CA and Visual Memory Delayed CA to accommodate the intercept differences identified in the earlier validation study.

**Figure 2:**
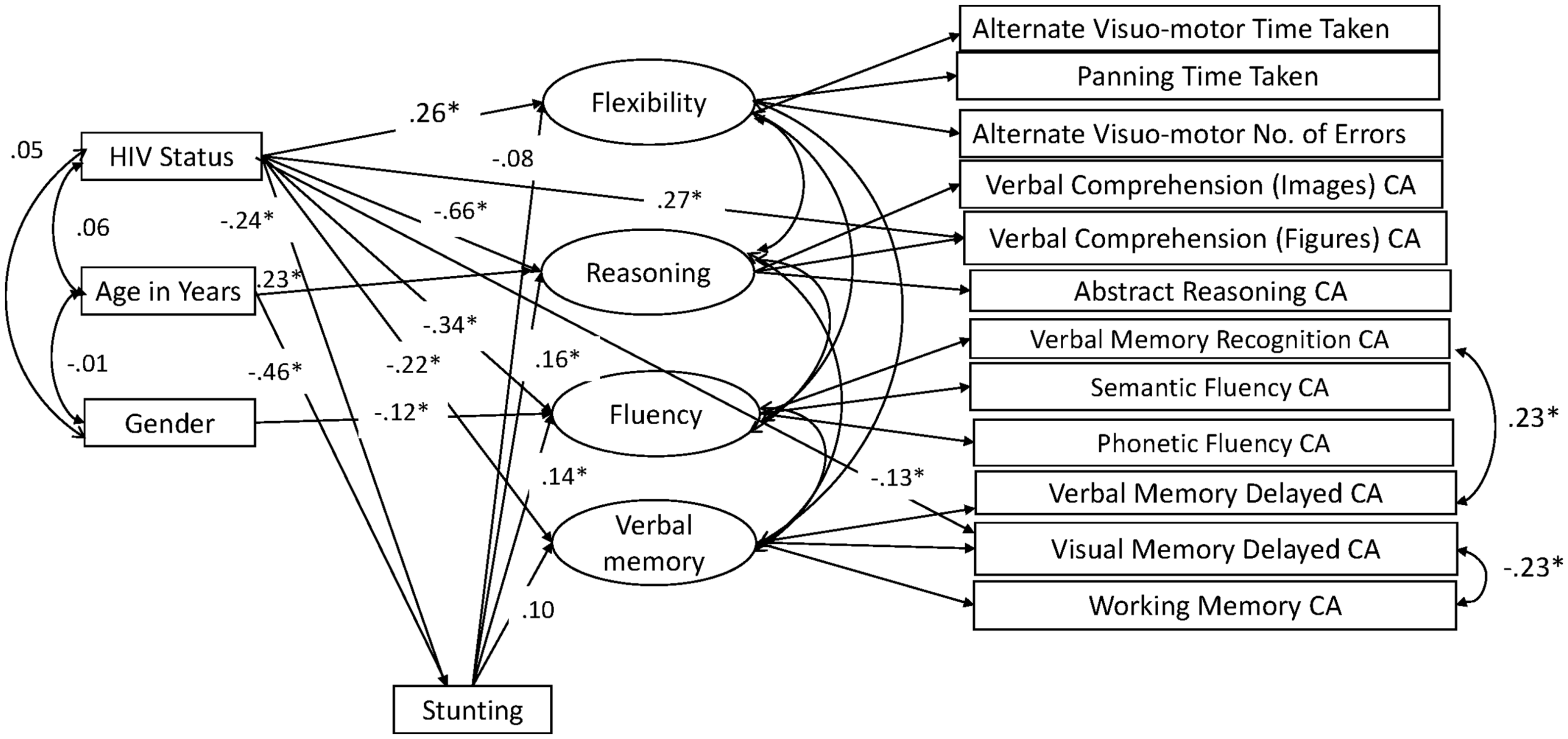
Height-for-age mediation model: significant paths in specification search. CA, Correct Answers.

Our previous paper on adapting and validating the BENCI in Kenyan children outlines the data cleaning process, including decisions in dealing with problematic data (19). The missing data pattern was not completely at random (Little’s MCAR test χ2 = 2455.2, DF = 1725, *p* < 0.001) but was not significantly related to factors that may have produced a missing pattern (19). Little’s MCAR test is also sensitive to non-normality, which might also play a role in the missing data pattern. However, we used data imputation in AMOS to check for modification indexes and calculate bootstrapped indirect effects. The modification indexes were used to check whether adding some paths would improve the model through a method of forward selection. Without overfitting the model too much, we decided *ad hoc* to add two residual covariances based on improper estimates of negative residual variances and modification indices. Residuals of Verbal Memory Recognition and Verbal Memory Delay were positively correlated, arguably due to the use of the same items across these indicators. The other residual covariance between Visual Memory Delay and Working Memory was unexpected but implemented to improve model fit. No further adjustments in the model as shown in Figure 1 were made. Bootstrapping based on 1,000 samples was performed to determine the significance of the direct, indirect, and total effects as well as their standard errors. We also fitted a model in which effects of HIV, age, and gender were fully mediated by stunting and ran a specification search model (Figure 2) in AMOS using the model in Figure 1 to assess the robustness of the results. We compared the fit of the models to assess mediation by stunting. Model fit was evaluated using goodness of fit indicators where an excellent fitting model would have a non-significant Chi-square test, Tucker Lewis Index (TLI) ≥ 0.95, Comparative Fit Index (CFI) ≥ 0.95, and Root Mean Square Error of Approximation (RMSEA) ≤ 0.08 (51). The term ‘predictive effect’ is used in this study to refer the hypothesized direction or the arrows within the model and finding prediction does not preclude that other factors have a role in causality.

We used a sample size of 604 as calculated in our paper putting forth psychometric validity of BENCI^1^ (19).

## Results

### Socio-demographic results

The total sample mean age was 9.48 (SD = 1.31) and the mean stunting (HAZ) was −0.44 (SD = 1.38). The prevalence of stunting in the HIV-positive sample was 17.9% while in the HIV-negative sample was 3.9%. The mean height-for-age in males was −0.42 (SD = 1.30) while in females it was −0.47 (SD = 1.45). Females who were HIV positive had more stunting (mean − 0.94, SD = 1.51) than their HIV-negative counterparts and both HIV-positive and negative males^2^. The details of the sociodemographic indicators are presented in Table 1.

**Table 1:** Socio-demographic indicators of the study population.

Variables		HIV uninfected *N* (%)	HIV infected *N* (%)
Gender	Female	163 (49.4)	148 (54.0)
	Male	166 (50.3)	125 (45.6)
	Missing	1 (0.3)	1 (0.4)
Age in months (Mean ± SD)		117.2 ± 16.24	119.40 ± 14.63
Age in Years (Mean ± SD)		9.41 ± 1.37	9.56 ± 1.24
Nutrition	Weight in kg (Mean ± SD)	34.98 ± 7.12	32.27 ± 5.85
	Height in cm (Mean ± SD)	136.34 ± 8.00	133.02 ± 8.11
	Height -for -age z score (Mean ± SD)	−0.13 (1.28)	−0.82 (1.39)
	Not stunted (≥ −1.9 SD)	265 (80.3)	180 (65.7)
	Moderately Stunted (− 2.9 to-2.0 SD)	11 (3.3)	41 (15)
	Severely Stunted (≤ −3.0 SD)	2 (0.6)	8 (2.9)
	Missing	52 (15.8)	45 (16.4)

Z score indicators are as provided by the WHO Child Growth Reference standards for 5–19-year olds (48).

### Height-for-age mediation model

We tested a full model (Figure 1) in which HIV status, age, and gender predicted the four cognitive executive functioning factors, and stunting acted as (partial) mediator of these predictions. This model showed good fit in terms of RMSEA = 0.041, CFI = 0.966, and TLI = 0.947, while the exact fit formally rejected the model (*χ*^2^ = 154.29, DF = 77, *N* = 604, *p* < 0.001), probably because of sensitivity to minor (distributional) violations and the relatively large sample size. The standardized effects and their level of significance results are presented in Table 2 and their standard errors are given in the Supplementary Appendix. We also ran a full mediation model without direct paths from age, gender, and HIV status on the cognitive latent variables, but this model showed poor fit (RMSEA = 0.074, CFI = 0.869, TLI = 0.824, χ2 = 384.22, DF = 89, *N* = 604, *p* < 0.001), highlighting that stunting does not fully mediate the effects of HIV on cognitive outcomes. A specification search yielded a more parsimonious model (Figure 2) that fitted well (RMSEA = 0.038, CFI = 0.967, TLI = 0.953, χ^2^ = 158.73, DF = 84, *N* = 604, *p* < 0.001) and corroborated our proposed model albeit without the significant paths.

**Table 2:** Height-for-age model standardized effects.

Bootstrapped estimates	Standardized indirect effects				Standardized direct effects				Standardized total effects			
	HIV status	Age in years	Gender	Height-for-age	HIV status	Age in years	Gender	Height-for-age	HIV status	Age in years	Gender	Height-for-age
Height-for-age					−0.242*	−0.462*	−0.004	-	−0.242*	−0.462*	−0.004	–
Flexibility	0.020	0.038	0.000	–	0.255*	−0.013	0.051	−0.082	0.275*	0.024	0.051	−0.082
Verbal memory	−0.024*	−0.046	0.000	–	−0.212*	0.041	−0.016	0.100	−0.236*	−0.005	−0.016	0.100
Fluency	−0.033*	−0.063*	0.000	–	−0.338*	0.075	−0.133*	0.136*	−0.371*	0.012	−0.134*	0.136*
Reasoning	−0.038**	−0.072*	−0.001	–	−0.655*	0.245*	−0.021	0.157*	−0.692*	0.173*	−0.021	0.157*
Alternative visual motor number of errors	0.167**	0.015	0.031	−0.050	–	–	–	–	0.167*	0.015	0.031	−0.050
Planning Time Taken	0.079*	0.007	0.015	−0.023	–	–	–	–	0.079*	0.007	0.015	−0.023*
Verbal Memory Delayed Hits	−0.174*	−0.004	−0.012	0.074	–	–	–	–	−0.174*	−0.004	−0.012	0.074
Visual memory delayed hits	−0.147*	−0.003	−0.010	0.062	−0.126*	–	–	–	−0.272*	−0.003	−0.010	0.062
Working Memory Hits	−0.174*	−0.004	−0.012	0.074	–	–	–	–	−0.174*	−0.004	−0.012	0.074
Verbal memory recognition hits	−0.068*	0.002	−0.025*	0.025*	–	–	–	–	−0.068*	0.002	−0.025*	0.025*
Semantic fluency hits	−0.281*	0.009	−0.101*	0.103*	–	–	–	–	−0.281*	0.009	−0.101*	0.103*
Phonetic Fluency Hits	−0.282*	0.009	−0.102*	0.103*	–	–	–	–	−0.282*	0.009	−0.101*	0.104*
Verbal comprehension images hits	−0.391*	0.098*	−0.012	0.088*	–	–	–	–	−0.391*	0.098*	−0.012	0.088*
Verbal comprehension figures hits	−0.523*	0.131*	−0.017	0.118*	0.268*	–	–	–	−0.255*	0.131*	−0.017	0.118*
Abstract reasoning hits	−0.394*	0.098*	−0.012	0.089*	–	–	–	–	−0.394*	0.098*	−0.012	0.089*
Alternate visuo-motor time taken	0.275*	0.024	0.051	−0.082	–	–	–	–	0.275*	0.024	0.051	−0.082

* *p* ≤ 0.05; ** ≤ 0.001; Hits-Correct Answers.

### Direct effects

As expected by its effects on poor nutrition and cognitive development, HIV infection (coded as 1 = HIV+) had a significant direct effect on height-for-age (Z score with lower scores, more stunting) (*β* = −0.242, *p* < 0.002) and on all cognitive latent variables (reasoning, fluency, verbal memory, and flexibility). HIV also had a direct effect on Verbal Comprehension Figures Hits and Visual Memory Delayed Hits reflective of the uniform measurement bias we described earlier (19). Age significantly predicted height-for-age (*β* = −0.462, *p* = 0.004). Also, age showed a direct effect on reasoning (*β* = 0.245, *p* < 0.001), but we found little evidence of direct age effects on fluency, verbal memory, and flexibility.

There was no gender difference in height-for-age (*β* = 0.00, *p* = 0.927). While males averaged higher fluency scores than females (*β* = −0.133, *p* = 0.005), gender did not significantly predict performance in flexibility, verbal memory, and reasoning. As expected from earlier works on the negative impact of stunting on cognitive outcomes, height-for-age predicted both fluency (*β* = 0.136, *p* = 0.008) and reasoning (*β* = 0.157, *p* = 002).

### Mediation effects

As shown in Table 2 and Supplementary Appendix, we found three significant indirect effects due to stunting between HIV grouping and verbal memory (*β* = −0.024, SE = 0.013, *p* = 0.047), fluency (*β* = −0.033, SE = 0.013, *p* = 0.005), and reasoning (*β* = −0.038, SE = 0.013, *p* = 0.001). An additional analysis captured in Supplementary Appendix suggested that the non-significant indirect path for flexibility (*β* = 0.020, SE = 0.012, *p* = 0.100) could be due to low power.

Given the failure of gender as a variable to predict height-for-age, height-for-age did not mediate the relationship between gender and any of the cognitive latent variables. In addition, an exploratory analysis captured in the Supplementary Appendix highlighted that females who were HIV positive showed more severe stunting, but adding the interaction between gender and HIV status rendered the direct path of gender on fluency non-significant. We deliberate on this finding in the Supplementary Appendix section on interaction effect.

Height-for-age mediated the relationship of age with fluency (*β* = −0.063, SE = 0.023, *p* = 0.009) and reasoning (*β* = −0.072, SE = 0.023, *p* = 0.002). Total effects are reported in Table 2, while their standard errors are reported in Supplementary Table A2 of the Appendix. Figure 2 reports the results of the specification search. Sensitivity analyses that checked for specification errors in the model are reported in Supplementary Table A1 in the Appendix.

## Discussion

We studied the mediating effects of stunting and the predictive effects of HIV, age, and gender on cognitive outcomes in a sample of 604 Kenyan children, and found that fluency, verbal memory, and reasoning are functions that may need to be targeted for intervention in children who were stunted and HIV positive. Next, we discuss these findings in detail.

### Height-for-age effects

Similar effects of height-for-age on language were found in a recent study in Kenya (25). Our study confirms this earlier study in showing the persistent nature of cognitive impairment among children who are stunted. Children who are stunted when aged 2 and who later recovered from stunting, remain underperforming on cognitive tests aged 5 compared to children who were never stunted (38). Two cross-sectional studies among children older than 5 years have found that children with better HAZ have better performance in the cognitive tests (25, 26) and found that height-for-age mediates the prediction with age of reasoning, memory, language, executive functions, and motor ability.

Our finding that HIV directly contributes to stunting has also been found in other studies within Sub-Saharan Africa (26, 53, 54). Children living with HIV infection and those exposed to HIV yet uninfected have a higher prevalence of stunting than children who are neither infected nor exposed to HIV. Hence, being infected or having been born to a mother who is HIV positive poses a risk to normal child development (22, 32, 55). Moreover, children who are stunted and living with HIV or exposed to HIV are likely to have persistent stunting as they age (32).

Our findings of stunting increasing with age have been found in other studies (25) and are consistent with the notion that stunting often persists over age. A study looking at changes in height-for-age among children living with HIV and started ART around 8 years found that stunting reached its peak at 13 years for boys and 12 years for girls (36). After this age, stunting declined though more slowly in boys (at 13 years 50%, 15 years 48% and 18 years 31% stunted) than in girls (at 13 years 35%, 15 years 25% and 18 years 15% stunted) (36). Though there is a dearth of studies and indeed consensus on the exact age when HIV most directly leads to stunting, there are variable suggestions such as a study that showed male and females do not differ by age and stunting at ART initiation when aged around 8 years (36). However, stunting z scores start to dip as early as the first year of life among children living with HIV than those exposed but not infected (33). Another explanation for the strong effect is how stunting is calculated, i.e., as height relative to age z score.

### HIV effects

We found children without HIV to outperform their HIV infected counterparts in all domains of cognitive functioning, with up to 44% of reasoning performance variation due to HIV. Our findings are consistent with those of earlier studies showing that children living with HIV score poorly in tests of nonverbal cognitive abilities, executive function, processing speed, memory, planning, reasoning, working memory, and visual–spatial abilities (22, 27, 28), especially in advanced stages of the disease (27). Suboptimal cognitive functioning significantly impedes the wellbeing of children. For instance, adherence requires memory capabilities for learning new information, encoding, storing and retrieving it when required (56). Similarly, for teenagers negotiating for healthy lifestyles, reasoning becomes an important asset. Deficits in these cognitive domains caused by HIV thus hinder psychosocial, learning processes including wading through routine functions and activities of daily life.

### Mediation by stunting

An earlier study in Kenya found that stunting mediated the effect of age and years in school on executive function, language, and motor skills, but not on verbal memory (25). These results are consistent with the current study findings related to language comprehension.

There is a dearth of evidence in form of comparative studies for such mediation findings among lower school students/school-age children. Among younger and older cohorts, age of stunting onset and gender impact cognitive development among children with HIV (36) and without HIV infection (33). A study that followed up children from birth till 5 years found significant lower cognitive scores among those with early stunting onset (1–6 months) compared to those who were never stunted (at 60 months). The effect of stunting on cognitive performance, however, was no longer significant among those with late stunting onset (7–24 months after birth) although this might have been due to low power (31). We would therefore expect indirect effects of age and HIV on cognition among lower school students/school-age children due to persistent stunting.

The statistically non-significant findings of the indirect effect of HIV on flexibility *via* height-for-age in our study could be attributed to low power. However, we note that there are additional underlying factors that determine good cognitive functioning among children who are stunted, such as lack of parental stimulation and few learning opportunities, which could contribute to cognitive deficiencies (38, 55). Indeed parental stimulation among children who are stunted has been seen to improve performance in language and IQ tests (57), and such factors warrant more research in the future.

### Age differences

The prediction of reasoning based on age was expected as reasoning increases with age and height-for-age reflects a history of stunted growth. However, other studies among a community samples have not found age differences related to reasoning and memory in 6–8 year-olds though the narrow age range could lower correlations with age (26). Such age differences in cognitive functions are expected because some functions such as inhibition develop earlier and rapidly more than others that appear later on in development (39). In other cases, late school onset and repeating a grade may create spurious age differences in cognitive performance (58) or obscure aging effects. Repeating children either improve academic achievement (59) or experience a decline in cognitive performance (60, 61) depending on how long they were retained though repeating may also reflect an existing low cognitive ability (62) among other persistent psychosocial and academic challenges.

The age-wise trend for cognitive performance should be steep but we found non- significant age differences in some of the cognitive indicators. Aging effects on cognitive performance could have been obscured by other risk factors that were not included and controlled for in our analysis. The few risk factors may have underpowered the findings resulting in non-significant correlations with age.

### Gender differences

Gender has been found to be associated with risk of stunting among children aged below 5 years where having female gender was protective against stunting (31). Though our study did not reflect the same findings, the direction of our outcome is seen in other studies where girls were found to be more stunted than boys [although Intiful, Abdulai (63) found this difference to be non-significant].

Males in our study outperformed the females in fluency function, but no other gender gaps emerged. Earlier studies documented that males outperformed females in other cognitive functions such as visual–spatial ability though females have better scores than males in memory (64). Gender differences in cognitive function have been linked to school achievement with females performing better in languages though some studies have not found any differences in some subjects (37, 65). Whereas such outcomes may bring up confusion on which gender is need of a certain cognitive intervention, such findings should be interpreted with caution because studies have shown age related sex differences in cognitive maturation (34).

A study on underlying factors in gender differences may contribute to giving boys and girls equal opportunities in development may it be in improved school performance and increased earning potential.

## Limitations

Our study interrogated a few independent variables while additional socio-economic factors and other confounding factors could affect cognitive functioning alongside stunting, HIV, age, and gender. Including additional factors such as poverty caregiver socioeconomic status, children schooling and related factors, and children’s familiarity with technology such as iPads in future studies might shed further light on the mechanisms causing lower cognitive functioning in populations infected by HIV. There is a dearth of studies evaluating the interplay between technology familiarity and cognition in children living with HIV and stunting. However, technological tools have been associated with cognitive development depending on exposure and pre-existing cognitive deficits (66). Moreover, though the Kenyan government obligates all parents to send children to school, our findings on the level of cognitive performance could be confounded by factors such as absentia and repeating grades among other factors. Our study did not control for such educational factors.

Our study used a cross-sectional design that is less able to uncover when and how effects emerge. A longitudinal study would point out the exact point where the severity of HIV strongly predicts cognitive deficiency in interaction with other determinants of stunting.

Our cross-sectional study design and study assessments do not allow us to uncover cognitive development trends within the children, or their ability to cope with early functional deficits (67). In addition, in cross-sectional studies, we cannot see whether the older children at an earlier time- point differ from the younger children in our sample. Of note, is that even longitudinal studies may miss out on this learning/coping confounding effect. Therefore, in situations where a child may be seen as underperforming, for example, in reasoning, they may have developed alternate ways of making sense of their environment such as through memorization. Indeed, children of the same age group have been found to have different patterns of developing reasoning functions (40). We may also not adequately explain differences in cognitive functioning of children of the same age who are brought up in different cognitively stimulating environments. With age, it is important to consider differences between following up the same cohort over time (33, 36, 61) and studying at one time-point (34, 38). Another limitation encompasses the cohort we used. These study findings and implications were drawn from a community sample and school factors such as student-to-teacher ratio and resources available in public vs. private schools may not have been matched to the sample. A hospital sample may present different findings hence the implications should not be overgeneralised.

Using longitudinal case–control designs, future studies could consider trends in different cognitive functions as factors of the environment they grown in, compensatory mechanisms for deficits and neurological mechanisms. Whereas our study takes a cross-sectional approach with few predictors, it is equally important in reviewing pediatric HIV programmes and setting up stunting and cognitive interventions.

## Conclusion

As strides are made to mitigate and better manage HIV in children while reducing new infections, addressing stunting as well as its cognitive effects remain crucial, especially with the added burden of HIV (3). Stunting appears to play a role in the effects of HIV on cognitive domains. Our results point to the importance of integrating interventions that target reasoning, fluency, and verbal memory cognitive functions among children suffering from HIV infection and stunting. Nutrition programmes looking into reversing the effects of HIV on cognitive outcomes among lower school children in LMIC can tailor interventions targeting stunting. This is by targeting reasoning, fluency, and verbal memory and a wider set of cognitive functions that may need to be rehabilitated based on future research findings.

## Data availability statement

The raw data supporting the conclusions of this article will be made available by the authors, without undue reservation.

## Ethics statement

The studies involving human participants were reviewed and approved by Tilburg University’s School of Humanities Research Ethics Committee (REC# 2017/25) and the Kenyatta National Hospital/University of Nairobi Ethical Review Committee (P556/07/2016). Written informed consent to participate in this study was provided by the participants' legal guardian/next of kin.

## Author contributions

RM, JH, AA, MP-G, MK, and JW significantly contributed to the study conceptualization, analysis, and manuscript preparation. RM, AA, JH, and MK conceptualized the study. RM conducted the data collection, entry, and clean up as JH, AA, MP-G, MK, and JW verified the dataset. Data analysis was done by RM, JH, and JW as MP-G, AA, MK, JH, and JW reviewed the findings and gave suggestions on how best the study objectives could be met through analysis. RM wrote the first manuscript draft that was read and reviewed by JH, AA, MK, MP-G, and JW. All authors contributed to the article and approved the submitted version.

## Funding

This study was part of a larger study that was funded through a seed grant for early career researchers organized by Partnerships for Mental Health Development in Sub-Saharan Africa (PaM-D) (NIMH award number U19MH98718) and a 2017 institutional award by the Kenyatta National Hospital’s Research & Programs Department (KNH/R&P/23F/55/13). The funding institutions had no role in the study conceptualization, data collection, analysis, data interpretation and manuscript preparation. AA and RM are also supported by the Office Of The Director, National Institutes Of Health (OD), the National Institute Of Biomedical Imaging And Bioengineering (NIBIB), the National Institute Of Mental Health (NIMH), and the Fogarty International Center (FIC) of the National Institutes of Health under award number U54TW012089 (AA and Waljee AK). The content is solely the responsibility of the authors and does not necessarily represent the official views of the National Institutes of Health.

## Conflict of interest

The authors declare that the research was conducted in the absence of any commercial or financial relationships that could be construed as a potential conflict of interest.

## Publisher’s note

All claims expressed in this article are solely those of the authors and do not necessarily represent those of their affiliated organizations, or those of the publisher, the editors and the reviewers. Any product that may be evaluated in this article, or claim that may be made by its manufacturer, is not guaranteed or endorsed by the publisher.

## Footnotes

^1^Using the sample in the validity paper (19) we determined the power of our model given the sample size of 604, RMSEA of 0.08 to assess misfit (if a model does not fit, RMSEA >0.08) and a model with 77 degrees of freedom (df). Using the function SemPower.PostHoc in R (52), a sample size of *N* = 604 is associated with a power larger than >99.9% to reject a wrong model with DF = 77 with an amount of misspecification corresponding to RMSEA = 0.08 using Alpha = 0.05 (52).

^2^Females who were HIV positive had more stunting (mean − 0.94, SD = 1.51) than females who were HIV negative (Mean − 0.02, SD = 1.24), males who were HIV positive (Mean − 0.68, SD = 1.23) and males who were HIV negative (Mean − 0.23, SD = 1.32) (*F* (1, 503) = 3.89, *p* = 0.049).
