# Supplementary material for: The effects of height-for-age and HIV on cognitive development of school-aged children in Nairobi, Kenya: a structural equation modelling analysis
Source: Front Public Health. 2023 Jun 21;11:1171851. doi: 10.3389/fpubh.2023.1171851 (PMC10321405; doi:10.3389/fpubh.2023.1171851)
Supplement: Supplementary file 1 [file Presentation_1.pdf]

Appendix

Sensitivity Analysis

A model specification search using AMOS resulted in the same as model shown in figure 3 after 2,097,152 models were tried. A model specification search is recommended for detection and correction of specification errors so that the initial theory implied model can reflect the true population model cognizant to the study variables (1). The resulting models and their fit indicators are indicated in Appendix: Table A1. The best model goodness of fit indicators was excellent with RMSEA = .038, CFI = .967, TLI = .953, $\chi 2$ (84, n = 604) = 158.731, p < .001. The model showed the specification search did not reveal missing links.

Data Cleaning

With weight and height measurements, there were 43 respondents with default entries. These were converted to missing data. We then checked for outliers through a scatter plot and statistical evaluation of weight and height. Before deleting the outliers, we checked for the residuals of the regression of age on the measurements where we noted measurements with high standardized residual value, low effect size, and low p-value (2). We then checked the *z* scores that were beyond z=2 and also triangulated the scores against what would be expected in other participants. As per this evaluation, we did not discard any more weight and height entries.

Flexibility Power Analysis

With flexibility, though there is a direct significant path between HIV and flexibility (p = .001), when height-for-age mediates this relationship, the path becomes non-significant (p =.100) though the power of this mediation is 0.5. In calculating this power (3), we used N = 604, path HIV to stunting β = -.24, path stunting to Flexibility β = -.08, path Flexibility to HIV β = .26 and alpha = .05 (3). This suggests that height-for-age does not fully mediate the relationship between HIV and flexibility though there is a 50% chance that we missed the indirect path if one exists in the population. Therefore, power could have been an issue.

Interaction Effect

Our study found no significant gender effects on stunting yet other studies have found stunting to be significantly higher in males compared to females (4, 5). We therefore set out to find out whether the relation between HIV and stunting was different for genders. We reran the path model again but this time added an interaction dummy variable for HIV and gender. The results showed a direct effect of the interaction variable on height-for-age (β = -.173, < .05) showing that a child that was female and HIV positive was most likely to be stunted. The only path that changed in the full model after adding the interaction between gender and HIV was that the gender effect on fluency which became non-significant (β = -.054, p = .362).

With flexibility, though there is a direct significant path between HIV and flexibility (p = .001), when height-for-age mediates this relationship, the path becomes non -significant (p =.100) though the power of this mediation is 0.5. In calculating this power (3), we used N = 604, path HIV to stunting β = -.24, path stunting to Flexibility β = -.08, path Flexibility to HIV β = .26 and alpha = .05 (3). This suggests that height-for-age does not fully mediate the relationship between HIV and flexibility though there is a 50% chance that we missed the indirect path if one exists in the population. Therefore, power could have been an issue

Table A1: Specification Search Stunting Mediation Models

Mo del	Name	Par ams	Df	C	C – df	AI C 0	BC C 0	BI C 0	C/ df	P	Not es		RMS EA	C FI 1	CFI 2

132	Unconstrained	52	84	158.73	74.73	0.00	0.00	11.65	1.89	0.00			0.04	0.97	0.96 142
142	Unconstrained	53	83	157.00	74.00	0.27	0.33	16.32	1.89	0.00			0.04	0.97	0.97
143	Unconstrained	53	83	157.25	74.25	0.52	0.58	16.57	1.89	0.00			0.04	0.97	0.96
112	Unconstrained	50	86	163.34	77.34	0.61	0.49	3.45	1.90	0.00			0.04	0.97	0.96
152	Unconstrained	54	82	155.52	73.52	0.79	0.90	21.24	1.90	0.00			0.04	0.97	0.97
122	Unconstrained	51	85	161.66	76.66	0.93	0.87	8.17	1.90	0.00			0.04	0.97	0.96
123	Unconstrained	51	85	161.86	76.86	1.13	1.07	8.37	1.90	0.00			0.04	0.97	0.96
133	Unconstrained	52	84	159.95	75.95	1.22	1.22	12.87	1.90	0.00			0.04	0.97	0.96
124	Unconstrained	51	85	162.14	77.14	1.41	1.35	8.65	1.91	0.00			0.04	0.97	0.96
134	Unconstrained	52	84	160.18	76.18	1.44	1.44	13.09	1.91	0.00			0.04	0.97	0.96

Table A2: Height-for-age Model Standardized Errors

Standard Errors	Standardized Indirect Effects				Standardized Direct Effects				Standardized Total Effects			
	HIV Status	Age in years	Gender	Height-for-age	HIV Status	Age in years	Gender	Height-for-age	HIV Status	Age in years	Gender	Height-for-age
Height-for-age	-	-	-	-	0.034	0.032	0.035	-	0.034	0.032	0.035	-
Flexibility	0.012	0.023	0.003	-	0.039	0.045	0.038	0.049	0.036	0.038	0.038	0.049
Verbal Memory	0.013	0.024	0.004	-	0.048	0.055	0.045	0.051	0.048	0.047	0.045	0.051
Fluency	0.013	0.023	0.005	-	0.045	0.053	0.045	0.049	0.043	0.048	0.045	0.049
Reasoning	0.013	0.023	0.006	-	0.042	0.048	0.042	0.047	0.040	0.042	0.042	0.047
Alternative Visual Motor Number of Errors	0.024	0.023	0.023	0.030	-	-	-	-	0.024	0.023	0.023	0.030
Planning Time Taken	0.014	0.011	0.011	0.015	-	-	-	-	0.014	0.011	0.011	0.015
Verbal Memory Delayed Hits	0.036	0.035	0.033	0.038	-	-	-	-	0.036	0.035	0.033	0.038
Visual Memory Delayed Hits	0.031	0.030	0.028	0.032	0.035	-	-	-	0.036	0.03	0.028	0.032
Working Memory Hits	0.037	0.035	0.033	0.038	-	-	-	-	0.037	0.035	0.033	0.038
Verbal Memory Recognition Hits	0.020	0.009	0.011	0.012	-	-	-	-	0.020	0.009	0.011	0.012
Semantic Fluency Hits	0.034	0.036	0.034	0.037	-	-	-	-	0.034	0.036	0.034	0.037

Standard Errors	Standardized Indirect Effects				Standardized Direct Effects				Standardized Total Effects			
Phonetic Fluency Hits	0.035	0.036	0.034	0.038	-	-	-	-	0.035	0.036	0.034	0.038
Verbal Comprehension Images Hits	0.029	0.024	0.024	0.027	-	-	-	-	0.029	0.024	0.024	0.027
Verbal Comprehension Figures Hits	0.092	0.030	0.032	0.039	0.097	-	-	-	0.037	0.03	0.032	0.039
Abstract Reasoning Hits	0.033	0.026	0.024	0.028	-	-	-	-	0.033	0.026	0.024	0.028
Alternate Visuo-motor Time Taken	0.036	0.038	0.038	0.049	-	-	-	-	0.036	0.038	0.038	0.049

References

1. Schumacker RE. Teacher's Corner: Conducting Specification Searches With Amos. Structural Equation Modeling: A Multidisciplinary Journal. 2006;13(1):118-29.
2. Maina R, He J, Abubakar A, Perez-Garcia M, Kumar M, Wicherts J. Psychometric Evaluation of the Computerized Battery for Neuropsychological Evaluation of Children (BENCI) among School Aged Children in the Context of HIV in an Urban Kenyan Setting. BMC Psychiatry. 2022;PREPRINT (Version 1) available at Research Square:In press.
3. Kenny DA. MedPower: An interactive tool for the estimation of power in tests of mediation [Computer software] 2017 [Available from: https://davidakenny.shinyapps.io/MedPower/.
4. Khan T, Khan REA, Raza MA. Gender Analysis of Malnutrition: A Case Study of School-Going Children in Bahawalpur. Asian Development Policy Review. 2015;3(2):29-48.
5. Astatkie A. Dynamics of stunting from childhood to youthhood in Ethiopia: Evidence from the Young Lives panel data. PLOS ONE. 2020;15(2):e0229011.
